# Sourdough Bread and Baker’s Yeast Bread Affect Gut Symptoms and Targeted Gut Microbial Taxa Differently: A Randomised Controlled Trial

**DOI:** 10.3390/nu18172854

**Published:** 2026-09-01

**Authors:** Solveig I. N. Watters, Christine Henriksen, Mia Gjøvik, Lisa Garnweidner-Holme, Marius Pihl Frederiksen, Stephanie Jonassen, Ina Ravnanger, Mari C. W. Myhrstad, Vibeke H. Telle-Hansen

**Affiliations:** 1 Department of Nursing and Health Promotion, Faculty of Health Sciences, Oslo Metropolitan University, 0130 Oslo, Norway; solveigi@oslomet.no (S.I.N.W.); miagjovik@outlook.com (M.G.); lgarnwei@oslomet.no (L.G.-H.);; 2 Department of Nutrition, Institute of Basic Medical Sciences, Faculty of Medicine, University of Oslo, 0317 Oslo, Norway; christine.henriksen@medisin.uio.no; 3 Mesterbakeren AS, 0552 Oslo, Norway; marius.pihl.fredriksen@idun.no (M.P.F.); stephanie.jonassen@mesterbakeren.no (S.J.); inaravnanger@hotmail.com (I.R.); 4 Intelligent Health, Faculty of Health Sciences and Faculty of Technology, Art and Design, Oslo Metropolitan University, 0130 Oslo, Norway

**Keywords:** sourdough bread, baker’s yeast bread, fermentation, whole-grain, dietary fibre, gut microbiota

## Abstract

**Background/Objectives:** The intake of bread has been associated with gastrointestinal discomfort in some individuals. The primary aim of this study was to investigate gut symptoms upon consuming sourdough bread compared to baker’s yeast bread. Secondary endpoints were changes in targeted gut microbial taxa, micronutrient levels, and metabolic markers. **Methods:** This randomised, controlled, double-blind cross-over study included twenty healthy participants. Sourdough or baker’s yeast bread was consumed for one week before crossing over. Before and after the interventions, participants reported gut symptoms, and they also provided blood samples to analyse micronutrients, metabolic markers, and faecal samples for targeted gut microbial taxa analyses. **Results:** The breads were made of whole grain wheat with similar macronutrients content. The sourdough bread contained less fructose than the baker’s yeast bread. The consumption of sourdough bread caused a significant reduction in the diarrhoea sub-dimension score (*p* = 0.016) and a borderline significant reduction in the overall GSRS-IBS score compared to baker’s yeast (*p* = 0.051). Several gut bacteria were significantly increased after intake of sourdough bread compared to baker’s yeast bread: *Alistipes putredinis* (*p* = 0.029), *Alistipes shahii* (*p* = 0.015), *Bifidobacterium adolescentis* (*p* = 0.042), *Bifidobacterium longum* (*p* = 0.010) and *Bifidobacterium longum subspecies longum* (*p* = 0.021). There was a significant correlation between changes in diarrhoea score and changes in *Alistipes shahii* (r= −0.353, *p* = 0.025). **Conclusions:** This exploratory cross-over study did not demonstrate a statistically significant difference in the overall GSRS-IBS score between the two breads. A nominal difference in the diarrhoea sub-dimension and selected targeted microbial taxa was observed between the two breads, but these findings require further investigations.

## 1. Introduction

The health beneficial effects linked to a high intake of dietary fibre are well established and include decreased risk of non-communicable diseases (NCDs), such as cardiovascular diseases, cancers, gastrointestinal disorders, and type 2 diabetes [1,2]. Whole grain is high in fibre, including fructans (fructooligosaccharides (FOS) and inulin) and galactans (galactooligosaccharides (GOS)). These fibres are utilised by beneficial bacteria and are considered to have prebiotic effects [3,4,5,6]. Despite these advantages, dietary fibre intake in western European countries continues to fall below recommended levels [7]. Bread represents a substantial source of dietary fibre in these countries [7] and is an important source of dietary fermentable oligosaccharides, disaccharides, monosaccharides, and polyols (FODMAPs). This is mainly due to the fructan content found in cereal flours [8]. FODMAPs are not hydrolysed in the small intestine but pass into the terminal ileum and the proximal colon, where they are rapidly fermented by gut microbiota [9]. Dietary restriction of FODMAPs is shown to alleviate gut symptoms due to decreased gut microbiota fermentation [10,11]. Consequently, individuals who experience gastrointestinal symptoms are increasingly avoiding high-FODMAP foods, such as bread [9,10].

Sourdough bread has been proposed as a potential alternative for those who restrict bread intake due to its lower FODMAP content [12]. Sourdough is characterised by a complex microbial community, which is mainly represented by lactic acid bacteria and several strains of wild yeast [11,13]. These microorganisms ferment the carbohydrates in the dough, producing carbon dioxide, which makes the sourdough bread rise. The unique activities of these microorganisms significantly influence the extent to which FODMAPs are broken down during fermentation and also contribute to the bread’s distinct flavour and texture [13,14]. Commercially made yeast, frequently referred to as baker’s yeast, is the primary leavening agent in modern baking due to its convenience and rapid fermentation time. Baker’s yeast mainly consists of a few strains of the yeast *Saccharomyces cerevisiae,* which does not provide the same complex microbial activity found in sourdough [11]. Therefore, compared to baker’s yeast, sourdough fermentation may have the potential to substantially degrade FODMAPs in the bread [11,13]. Sourdough bread may therefore be more tolerable and a suitable alternative for those who refrain from bread due to gastrointestinal symptoms. Additionally, sourdough fermentation is suggested to influence other nutritional features of bread as well, such as lowering the glycaemic response and improving nutrient availability [11,12,13,15,16]. However, many studies investigating health effects of sourdough are mainly in vitro studies [17]. In human studies investigating the impact of sourdough bread on gastrointestinal symptoms and gut microbiota, the results have been conflicting [18,19,20,21,22,23,24]. This may be due to other components than sourdough fermentation *per se* or that the effect of sourdough bread as part of a diet is too small to be measured in humans [18,21,22,25]. This study aimed to investigate whether the intake of sourdough bread compared to baker’s yeast bread may cause fewer gut symptoms in individuals who are sceptical towards consuming bread and/or portray self-reported mild to moderate gut symptoms upon bread consumption. In addition, this study aimed to investigate whether the intake of sourdough compared to baker’s yeast bread may change specific gut microbial taxa and beneficially affect circulating levels of micronutrients and metabolic markers.

## 2. Materials and Methods

### 2.1. Study Design and Subjects

**Figure 1:**
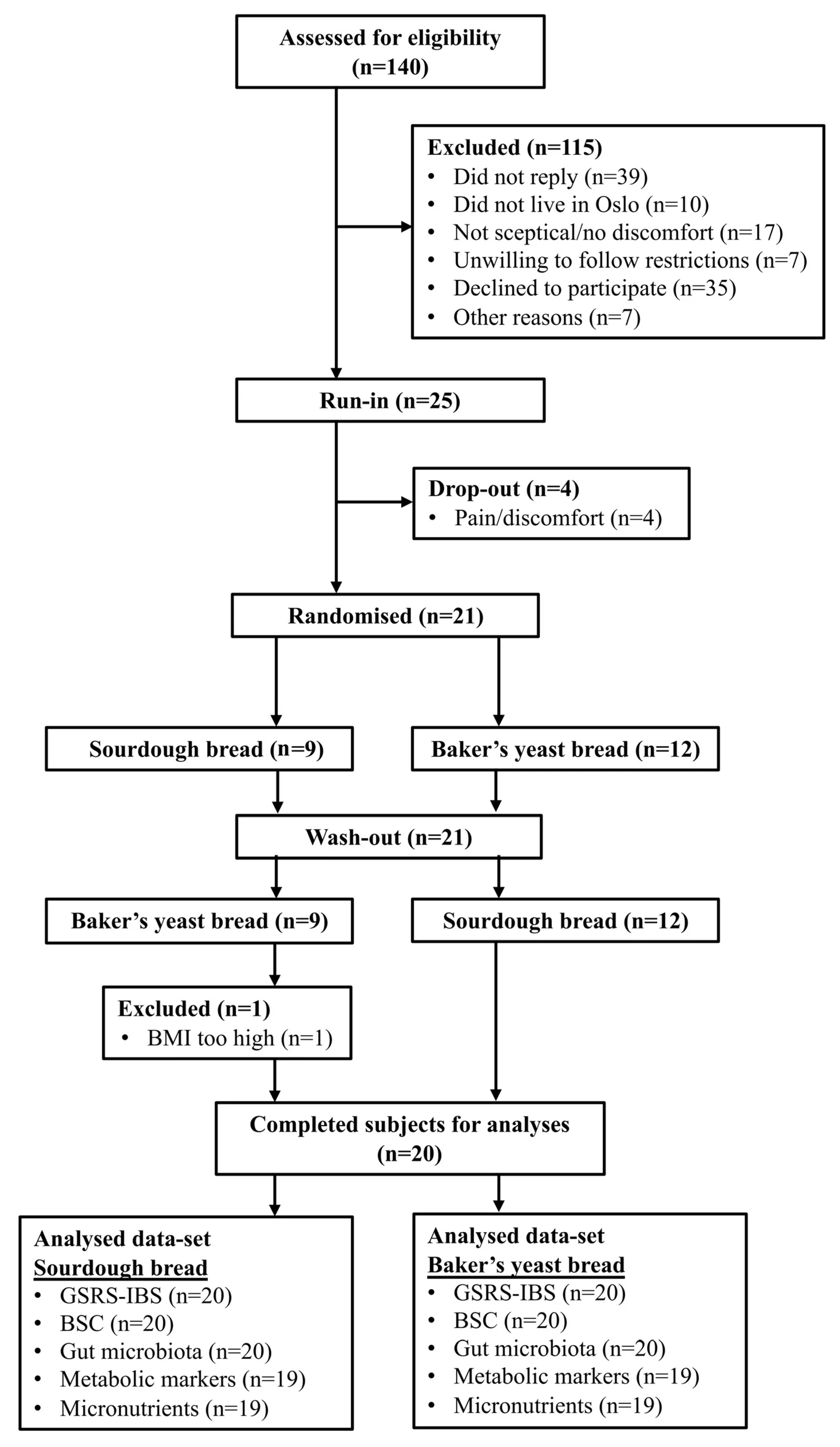
Flow chart of the participants and overview of the data analyses. Abbreviations: BMI: Body mass index; BSC: Bristol stool chart; GSRS-IBS: Gastrointestinal symptom rating scale for irritable bowel syndrome.

Healthy volunteers who were sceptical towards consuming bread and/or portrayed self-reported mild to moderate gut symptoms upon bread consumption were recruited to participate in this remotely conducted randomised, controlled, double-blind, cross-over study. Volunteers were recruited via the Oslo Metropolitan University (OsloMet) website and via social media from September to December 2020. The study included healthy adults (18 to 65 years) with a body mass index (BMI) between 18.5 and 27 kg/m^2^, living in Oslo and the surrounding area. The average BMI in the Norwegian population is 26.5 kg/m^2^ for men and 25.6 kg/m^2^ for women [26], and a BMI less than 27 kg/m^2^ was therefore chosen as one of the inclusion criterion. To be included, the participants had to refrain from consuming bread and bread products other than those provided in the study as well as probiotic products (i.e., Biola, Activia), fermented foods (i.e., Kimchi, Kombucha), dietary supplements, and hormonal treatments (except oral contraceptives) starting four weeks before and during the study. Other exclusion criteria included chronic metabolic diseases (e.g., diabetes, cardiovascular disease, and cancer), food allergies or intolerances towards products in the study, treatment with antibiotics during the previous three months and throughout the study, blood donations the previous two months and during the study, pregnancy or lactation, planned weight reduction or 5% weight change in the previous three months, high alcohol consumption (>40 g per day), and tobacco use. Of the 140 inquiries, twenty-five participants fit the inclusion criteria and signed the consent form to commence run-in (Figure 1). Four participants dropped out during run-in because of stomach pain and discomfort, and one participant was excluded at the end because of weight increase and BMI > 27 kg/m^2^, leaving twenty participants complete for analyses.

**Figure 2:**
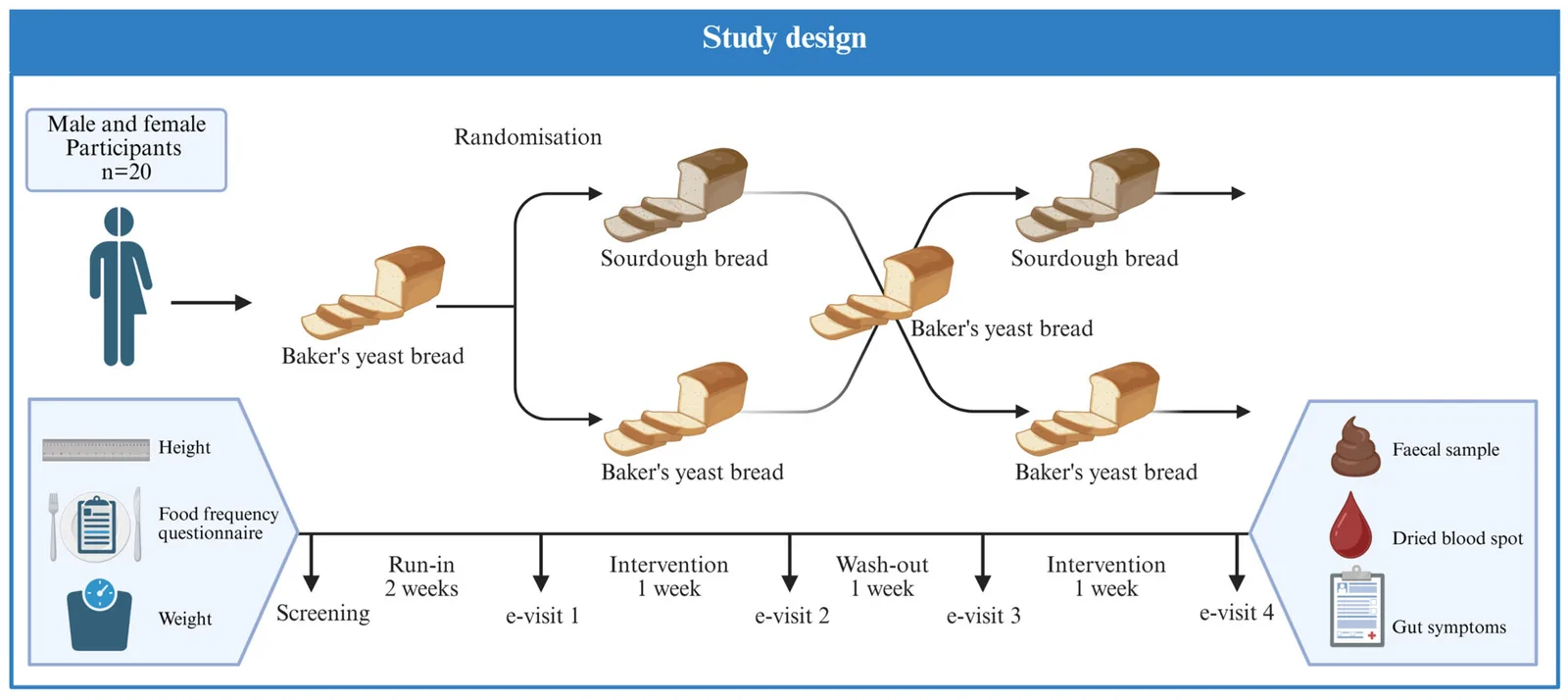
Study design of the double-blind, randomised, controlled cross-over study in which twenty participants consumed sourdough bread or baker’s yeast bread for one week, which was separated by a 1-week wash-out period. The participants consumed baker’s yeast bread in the run-in and wash-out periods. The study was performed remotely with five e-visits (screening, e-visit 1–4) and delivery of products and/or sampling kits at four time points. Faecal samples (gut microbiota) and fasted blood samples (micronutrients, metabolic markers) were collected by the participants and returned to OsloMet by regular mail in ready made envelopes, and gut symptoms (GSRS-IBS, BSC) were assessed at e-visits 1–4 (before and after both intervention periods). In addition, the participants completed a food frequency questionnaire (FFQ) during screening, and self-reported anthropometrics measurements were collected during screening (hight and body weight) and at e-visits 1–4 (body weight). The figure was made with BioRender.

Due to COVID-19, the study was conducted remotely, and all communication with the participants was carried out digitally via e-visitation (zoom-meetings), e-mail and telephone. Screening and health examination were conducted via e-visit and included self-reporting of pre-existing medical history, height, body weight and BMI. Before the start of the study, all participants received and completed a food frequency questionnaire (FFQ) designed to register their habitual diet during the past year [27]. The study endured for five weeks and commenced with a two-week run-in (Figure 2). After the run-in period, the participants were randomised into one of two interventions, consuming either sourdough bread or baker’s yeast bread for one week before crossing over to the other intervention, which was separated by a 1-week wash-out period. All participants consumed baker’s yeast bread during the run-in and wash-out periods.

At four time points throughout the study, the participants received a delivery containing equipment and instructions for data collection, ready-made return envelopes and study breads. Each intervention period included two e-visits (before and after), adding up to a total of five e-visits, including screening (screening and e-visits 1–4). During e-visits 1–4, the participants submitted responses to two forms online, the Gastrointestinal symptom rating scale for irritable bowel syndrome (GSRS-IBS) and Bristol stool chart (BSC) measuring gastrointestinal symptoms and stool classification, respectively. The participants also collected faecal and blood samples (Dried blood spots (DBSs)) at home following instructions and returned them by regular mail in the ready-made envelopes after e-visits 1–4. Blood samples were collected after an overnight fast (≥12 h) in the morning of e-visits 1–4, and faecal samples were collected the day before e-visits 1–4. A Case Report Form (CRF) was completed during e-visits 1–4 to assess adherence to protocol and report possible changes in diet, physical activity, or health status. Additionally, we conducted individual interviews with the ten first participants to complete the study to investigate attitudes towards and experiences with sourdough bread compared to baker’s yeast bread, which was previously published [28].

### 2.2. Study Products

This study included bread baked with baker’s yeast (control) and bread baked with sourdough (experiment). The recipe for both study breads was developed by Mesterbakeren AS bakery, Oslo, Norway. Except for the leavening agent and duration, the recipes for the study breads were identical and matched in macro- and micronutrient content and contained 56% coarsely ground flour, 72 g of whole grains, and 11.2 g of fibre. The loaves of bread were prepared using the following ingredients: water, salt, whole wheat flour, and a leavening agent (sourdough or baker’s yeast). The sourdough mixture was fermented in a proving cabinet (Lillonord, Odder, Denmark) at 25 °C and stirred every two hours for 8 h before being kneaded in a planetary mixer and portioned into 750 g pieces. The sourdough bread was then shaped and transferred into loaf pans and placed in a proving cabinet holding 24 °C and 72% air humidity for approximately 14 h. The baker’s yeast bread was prepared identically except for the initial fermenting process. Additionally, the baker’s yeast bread was proofed at a temperature of 32 °C and for 60 min.

The breads were prepared by Mesterbakeren AS, packed in neutral packaging, and stored at −20 °C at OsloMet, campus Kjeller. The participants received a minimum of four frozen and pre-sliced breads delivered by courier each intervention week [28]. Slices of bread were standardised to approximately 11 mm, and each slice weighed 40 g +/− 3 g. Participants were instructed to consume at least five slices of bread daily (200 g). Participants were requested to record adherence to the protocol by registering the number of bread slices consumed daily. Eurofins (Eurofins Food & Feed Testing Norway AS, Moss, Norway) analysed the two types of study breads to determine total macronutrient content, including total fibre and the following FODMAP components: fructose, lactose, and fructans (inulin/FOS) and micronutrients including zinc, selenium, and iron.

### 2.3. Blinding

The study was double-blind; hence, neither participant nor researchers collecting data knew the treatment allocation. Participants were randomly assigned to start with either the baker’s yeast bread or the sourdough bread following a simple randomisation procedure. During the randomisation process, Excel was used to generate a random number between 1 and 50 for each ID number. Participants assigned odd numbers were placed in the experimental group, while those with even numbers were assigned to the control group. The breads were packed in neutral packaging and then delivered to the participants. The randomisation and packing of the bread were carried out by a researcher who was not involved in data collection, follow-up of participants or statistical procedures. The randomisation and treatment allocation were disclosed after all statistical analyses were completed. Blinding assessment was not performed during the study, but participants were invited to take part in a qualitative interview that included questions about the study breads at the end of the study period [28].

### 2.4. Measurements of Gastrointestinal Symptoms

Gastrointestinal symptoms were measured by digital versions of the GSRS-IBS and BSC forms [29,30] administered to the participants by e-mail during screening and e-visits 1–4. All answers were collected and stored in Services for Sensitive Data (Tjenester for sensitive data: TSD). The participants were asked to rate themselves on the GSRS-IBS retrospectively. The GSRS-IBS consists of 13 questions regarding abdominal pain or discomfort, including gas, diarrhoea, constipation, and symptoms such as the urgent need to have a bowel movement, trouble emptying the bowel, and unusual satiety patterns. Each question assesses symptoms using a 7-point Likert scale ranging from no symptoms (1 point), minor (2 points) or mild discomfort (3 points), then moderate (4 points) and moderately severe discomfort (5 points), severe discomfort (6 points) and very severe discomfort (7 points) and with an overall score range of 13–91 points [29]. The GSRS-IBS includes five sub-dimensions with respective score ranges: pain (2–14 points), bloating (3–21 points), constipation (2–14 points), diarrhoea (4–28 points), and satiety (2–14 points). The participants were also asked to classify their stools on the BSC from 1 to 7 [30]. Bristol types 1–2 are consistent with severe and mild constipation, 3–4 are normal, and 5 through 7 are coherent with diarrhoea. At screening, the median (25–75 percentile) GSRS-IBS total score was 24.00 (18.25–32.75) and BSC was 4 (3.5–5.0).

### 2.5. Blood Sampling

Capillary blood samples were collected by Dried Blood Spot (DBS) and analysed at a core facility (Vitas Analytical Services, Oslo, Norway). HbA1c, total cholesterol, triglycerides, c-peptide, iron, zinc, and selenium were analysed from DBS. A self-collection DBS kit containing the required equipment was delivered to the participants. After fasting overnight (≥12 h), the participants collected finger-prick blood samples, which were assisted by both oral and written instructions. Each participant was instructed to sanitise one finger with an isopropyl alcoholic swab before collecting capillary blood samples by finger-prick with a sterile lancet. The participants discarded the first drop of blood before completing two DBS cards, each with five drops of blood (250 μL). The participants were then instructed to let the cards dry at room temperature for at least two hours before packing the samples in an airtight aluminium bag containing a sachet of silica as desiccant material (Whatman Foil Bags, Whatman Inc., Florham Park, NJ, USA) and returning it by regular mail to OsloMet, campus Kjeller. The DBS samples were stored at −80 °C until dispatched to VITAS (Vitas Analytical Services, Oslo, Norway) for analysis.

### 2.6. Analysis of Blood Samples

Total cholesterol was quantified in DBS using gas chromatography-flame ionisation detection (GC-FID) (Agilent 7890A Gas Chromatograph System, Agilent Technologies, Palo Alto, CA, USA) and is given as mmol/L. Cholesterol was separated on a TR-FAME column (Thermo Scientific 30 m × 0.25 mm × 0.25 μm column). Triglyceride was quantified in DBS by liquid chromatography with ultraviolet detection (LC-UV) (Agilent 1260 Infinity Liquid Chromatograph System, Agilent Technologies, Palo Alto, CA, USA) and is given as mmol/L. The triglyceride sample was separated on a C18 column using water and methanol and detected at 590 nanometres (nm).

HbA1c was quantified in DBS using Pointe Scientific (Medtest, Canton, MI, USA) and is given as mmol/mol. The absorbance was read at 660 nm using a Spark 10M microplate reader (Tecan, Männedorf, Switzerland). C-peptide was quantified in DBS by sandwich enzyme-linked immunosorbent assay (ELISA) (The Ultrasensitive c-peptide kit, Mercodia AB, Uppsala, Sweden and Merck Millipore, Billerica, MA, USA, respectively) and is given as pmol/L. The absorbance was read spectrophotometrically at 450 nm using a Spark 10M microplate reader (Tecan, Switzerland). The micronutrients zinc, selenium and iron were quantified in DBS using liquid chromatography (LC, 1100 series Agilent Technologies, Waldbronn, Germany) inductively coupled plasma mass spectrometry (LC/ICP-MS (Agilent 7900 ICP-MS, Agilent Technologies, Tokyo, Japan) and are given as μg/mL (zinc/selenium) and umol/mol (iron).

### 2.7. Faecal Sampling and Analysis of Faecal Microbiota

Faecal samples were collected for analysis of targeted gut microbial taxa performed at a core facility (Bio-Me Microbiome Profiling, Oslo, Norway). Participants received cards containing squares of Whatman^®^ 903 filter paper on which to apply their stool samples along with a flushable toilet seat cover for sample collection (Fe-Col^®^, Col-group, Amsterdam, Netherlands), a lollipop stick applicator, gloves, an airtight foil bag (EBF (Eastern Business Forms), Mauldin, SC, USA) for storage and ready-made envelopes for returning the samples. Before sample collection, participants received oral and written instructions regarding how to collect the faecal sample with the equipment provided. Participants were instructed to collect the sample in the toilet seat cover, apply a thin layer of the stool onto the filter card using the lollipop stick applicator, and allow it to air dry at room temperature for at least two hours. After drying, the filter card should be placed in the foil bag containing a sachet of silica as a desiccant (Whatman Foil Bags, Whatman Inc.). Samples were collected the day before the e-visit and labelled with the date and ID numbers and stored at room temperature until they were returned by regular post. When received, samples were stored at −80 °C at OsloMet before being dispatched to Bio-Me (Bio-Me Microbiome Profiling, Oslo, Norway) for analysis.

The gut microbiota was analysed using a validated quantitative PCR method (Bio-Me’s Precision Microbiome Profiling PMP™, Oslo, Norway). This method employs custom-made OpenArray^®^ qPCR panels that targets 108 prevalent and well-known gut microbiota species, including 107 bacterial and archaeal species/subspecies, as well as one fungal genus (Candida) [31,32]. Three discs of filter paper, each 6 mm in diameter, were punched out from each sample filter card, and microbial DNA was extracted using a MagMAX™ Microbiome Ultra Nucleic Acid Isolation Kit (Thermo Fisher Scientific, Waltham, MA, USA) following the KingFisher™ Flex protocol (Thermo Fisher Scientific, Waltham, MA, USA). The bacterial cell walls were disrupted with a Star Beater (VWR, West Chester, PA, USA) at a frequency of 30 Hz for 2 min. The purified DNA was eluted in 200 µL of MagMAX™ Elution Buffer and quantified using the Quant-iT™ PicoGreen™ dsDNA Reagent (Thermo Fisher Scientific, Waltham, MA, USA) and an Infinite F200 Fluorescence Microplate Reader (Tecan Group AG, Männedorf, Switzerland). The sample microbiome DNA from the participants was analysed using the Precision Microbiome Profiling (PMP™, Bio-Me, Oslo, Norway), custom-made OpenArray^®^ qPCR panels for direct quantification of the targeted taxa. The analysis followed the default protocol on TaqMan^®^ OpenArray^®^ Real-Time PCR Plates (Thermo Fisher Scientific, Waltham, MA, USA). Absolute quantification, expressed as the number of genomic copies per µL, was calculated for each assay targeting specific bacterial species or subspecies by interpolation from standard curves derived from quantified reference isolates obtained from DSMZ (Leibniz Institute, Braunschweig, Germany) or ATCC (American Type Culture Collection, Manassas, VA, USA). Normalized quantification, specifically the number of genomic copies per nanogram of DNA, was calculated by dividing the absolute quantification for each target by the total DNA concentration in the sample, enabling between-samples comparisons. The normalised quantification of the 107 target bacteria was used in this study.

### 2.8. Statistical Analysis

The primary outcome measure was changes in gastrointestinal symptoms measured by GSRS-IBS as described in Clinical Trials (NCT04677881). Secondary endpoints were changes in stool classification measured by BSC, faecal gut microbiota, and capillary blood levels of c-peptide, total cholesterol, triglycerides, iron, zinc, and selenium. The sample size was determined based on the proportion of subjects included in a previous study that observed changes in gut symptoms related to FODMAP consumption [22]. The sampled metabolic markers, micronutrients, and gut bacteria species without a detectable level in at least 50% of the participants at each time point were discarded from further analyses. For the targeted gut microbial taxa measurements, each faecal sample was analysed for the abundance of a given set of 107 gut bacteria species. Of the 107 bacteria species, 45 were evident in at least 50% of the participants and included in the analyses. Participants with missing values before and after one of the interventions were excluded from the analysis. More specifically, one participant was excluded from the metabolic markers and micronutrient analyses, giving a total of n = 19. For participants with missing values at individual time points in the dataset, these values were replaced with the mean of the observations for each variable at the respective time point. Additionally, samples with concentrations less than the lower limit of quantitation (LLOQ) values were assigned the respective LLOQ divided by two (LLOQ/2) for that marker. Two values were replaced with an estimated value for the marker triglyceride, and one value was replaced with LLOQ/2 for HbA1c. Furthermore, two GSRS-IBS forms (different participants) were missing and were also replaced with estimated values.

Given the sample size of the current study, non-parametric tests were used for statistical analyses of the fasted blood samples (micronutrients, metabolic markers), and gut symptoms (GSRS-IBS and BSC) and values are presented as median and 25–75th percentiles. The Wilcoxon signed-rank test was used to analyse differences between interventions (sourdough and baker’s yeast bread) and within each intervention period, specifically before and after the intake of sourdough and baker’s yeast bread. The Mann–Whitney test was used to analyse differences between responders and non-responders.

**Figure 3:**
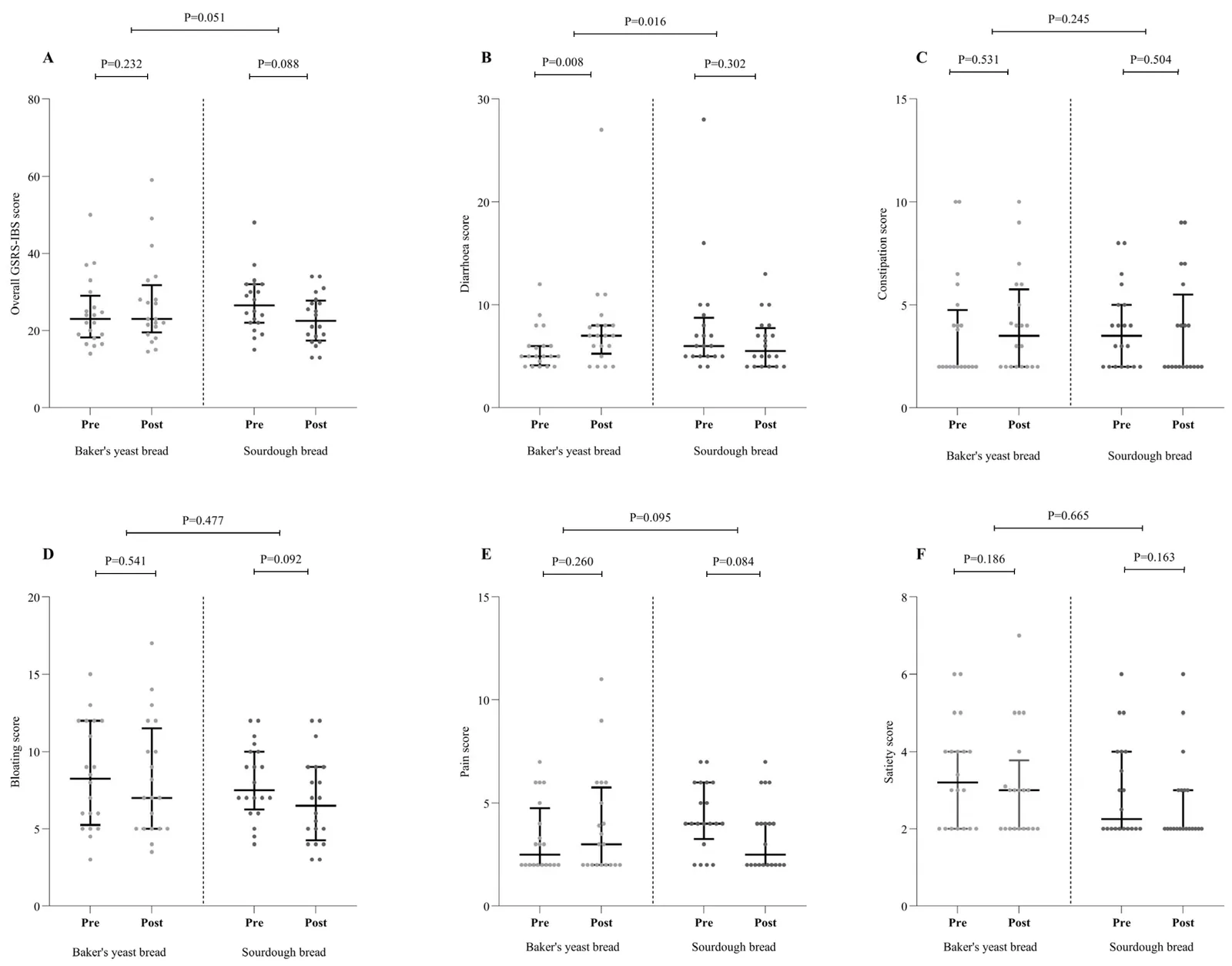
Scatter dot plot of GSRS-IBS scores, including (**A**) overall GSRS–IBS (range: 13–91) and sub-dimensions: (**B**) diarrhoea (score range: 4–28), (**C**) constipation (score range: 2–14), (**D**) bloating (score range: 3–21), (**E**) pain (score range: 2–14), and (**F**) satiety (score range: 2–14) before and after one week of eating baker’s yeast bread or sourdough bread (n = 20). Overlaid horizontal bars indicate median and 25–75th percentiles. Pre and post indicate before and after the interventions. Statistical differences between and within interventions were analysed using the Wilcoxon signed-rank test, and significance levels are defined as *p* < 0.05. The figure was made with GraphPad.

**Figure 4:**
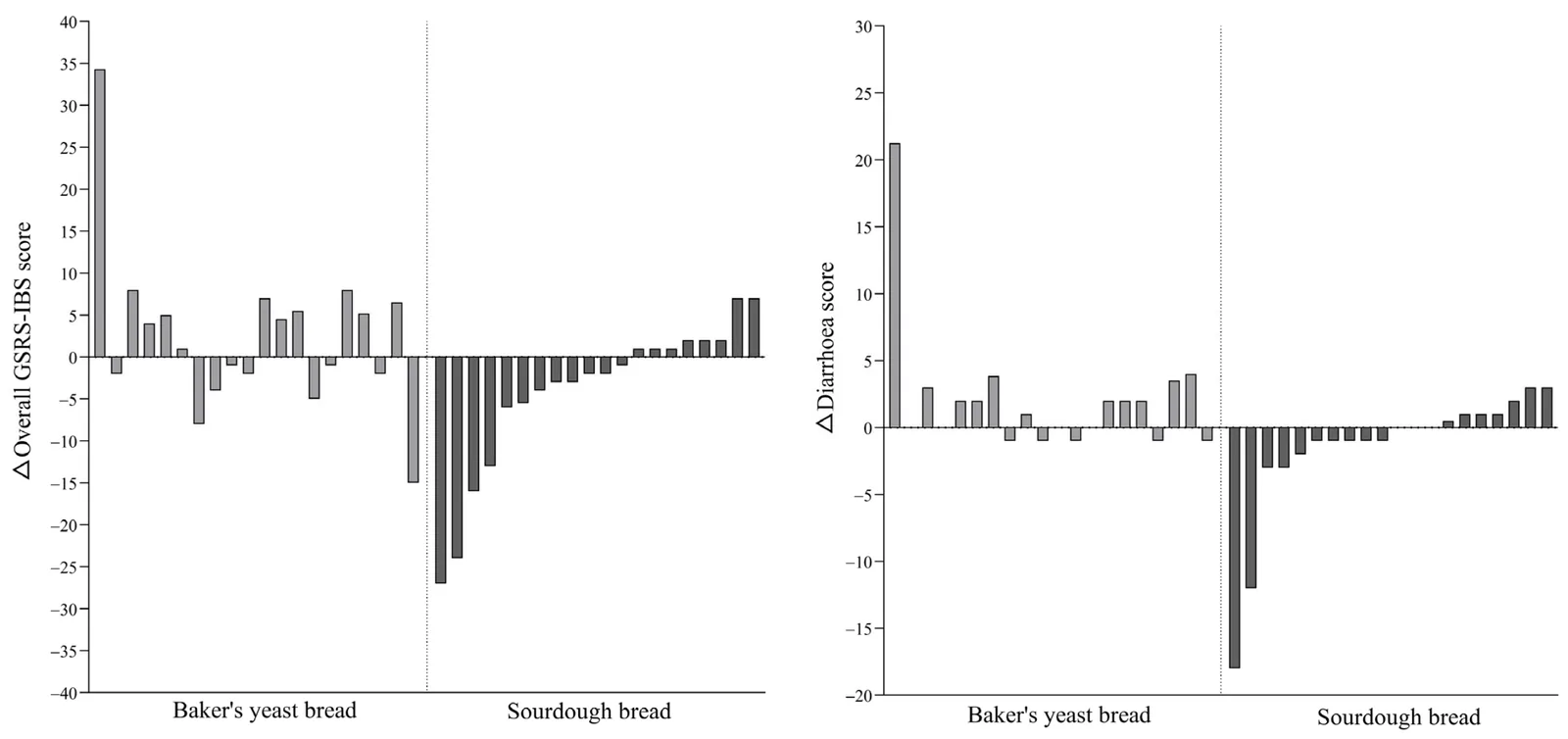
Individual changes in gut symptoms. Each bar represents the individual change in overall GSRS-IBS score and diarrhoea score after one week of consuming baker’s yeast bread and sourdough bread (n = 20). Figures were made with GraphPad.

**Figure 5:**
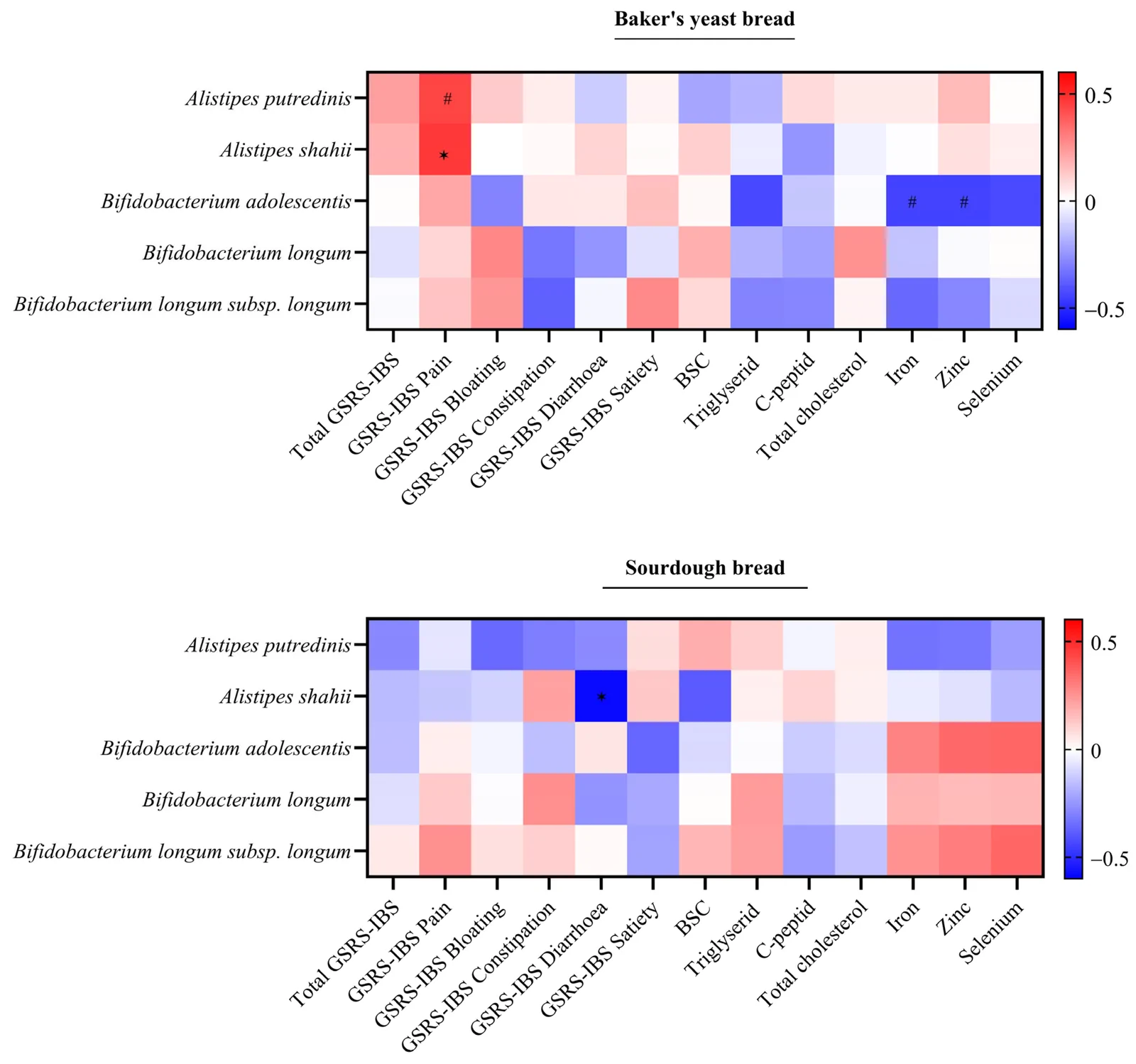
Relationship between changes in gut bacteria species and gut symptoms, metabolic markers and micronutrient levels. The five gut bacteria species that changed after intervention with sourdough bread compared to baker’s yeast bread (as shown in Table 5) are included in the analyses. The bacterial taxa are sorted from negative (blue) to positive (red) correlations assessed by Spearman’s rho correlation. * Indicates *p*-value < 0.05, while # indicates a borderline significant p-value between 0.05 and 0.06. Abbreviations: BSC: Bristol Stool Chart, c-peptide: connecting peptide, GSRS-IBS: Gastrointestinal Symptom Rating Scale for Irritable Bowel Syndrome. The figure was made with GraphPad.

The normalised quantification number of the 45 species was logarithmically transformed with base 2 (Log_2_) to reduce the skewness of the original data, and analyses were therefore conducted with parametric tests. The Log_2_ ratio (Log_2_ values post/pre interventions and sourdough bread/baker’s yeast bread) was calculated for the statistical analysis of the gut microbiota. A paired samples *t*-test assessed differences between the interventions (sourdough and baker’s yeast bread) as well as within each intervention period (specifically before and after intake of sourdough bread and baker’s yeast bread) based on the Log_2_ ratio. Associations between significant changes in gut microbiota and gut symptoms and biomarkers in blood samples (metabolic markers and micronutrients) were assessed with Spearman’s rho correlation. *p* < 0.05 was considered statistically significant for all statistical analyses, while *p* = 0.05–0.07 was considered a trend. The data were processed in Microsoft 365 MSO (version 2302) and statistically analysed in IBM SPSS statistics (version 29). Figure 1 and Figure 2 were designed using Power Point (version 2408), and Figure 3, Figure 4 and Figure 5 were designed using GraphPad Prism 9 (version 9.4.1).

## 3. Results

### 3.1. Baseline Characteristics

Twenty participants (five males and 15 females) completed this randomised, controlled, double-blind, cross-over study, and the baseline characteristics of the participants are shown in Table 1. The participants were normal-weight (median BMI of 22.2 kg/m^2^) adults (median age of 28 years) with HbA1c, total cholesterol, triglycerides, and c-peptide levels within the normal range (Table 1). Information about the participants’ habitual diets, including their nutritional intake levels, was collected by FFQ and is presented in Table 2. Before the study, the median (25–75th percentiles) daily energy intake was 9227 kJ (8059–10836 kJ). The carbohydrate intake was below the recommendation (45–60 E%) with 42.4 E%. The protein and fat intake were within the recommendations (10–20 E% and 25–40 E%, respectively) with 15.6 E% and 35 E%, respectively. The saturated fat intake was higher than recommended (<10 E%) with 12.4 E%. Before the start of the study, the participants reported a daily intake of 102.3 g of bread (approximately 2.5 slices), 53.3 g whole grain and 29.5 g of fibre in their habitual diet (Table 2).

### 3.2. Intervention Breads

The nutritional composition of the two study breads offered to the participants is presented (per 100 g) in Table 3. The study breads were based on identical recipes (except for the leavening agent and duration) and were made of whole grain wheat flour and contained similar amounts of macro- and micronutrients (iron, zinc, and selenium). The amount of fibre (6.5 g/100 g and 6.3 g/100 g in the sourdough and the baker’s yeast, respectively) was also similar between the two breads. The intake of analysed FODMAPs from the sourdough bread was 1.90 g/day, while it was 2.62 g/day from the baker’s yeast bread. The sourdough bread contained 0.11 g fructose, 0.80 g inulin/FOS, and <0.04 lactose per 100 g, while the baker’s yeast bread contained 0.87 g fructose, 0.40 g inulin/FOS, and <0.04 g lactose per 100 g (Table 3). The participants’ compliance with the study protocol was calculated to be 94% for both the sourdough bread and baker’s yeast bread. Participants reported consuming a median intake of 5 slices of bread daily during the sourdough bread and baker’s yeast bread intervention weeks.

### 3.3. Gut Symptoms

The primary endpoint of this study was to investigate whether the intake of different types of bread could influence gastrointestinal symptoms. There was a reduction in overall GSRS-IBS score of 2 points (25–75th percentiles: −5.9–+1.8) after consuming sourdough bread compared to an increase of 2.5 points (−2.0–+6.3) after consuming baker’s yeast bread. The difference between the groups did not reach statistical significance (*p* = 0.051). There was a significant reduction in the GSRS-IBS diarrhoea sub-dimension score of 0.5 points (−1.8–+1.0) after the sourdough intervention compared to a 1.5 points (−0.8–+2.8) increase after the baker’s yeast intervention (*p* = 0.016) (Figure 3). Furthermore, the GSRS-IBS diarrhoea sub-dimension score was found to be significantly increased after consuming baker’s yeast bread (*p* = 0.008). When looking into the individual changes, we found that while some participants experienced a beneficial effect from eating sourdough bread with a corresponding negative effect of baker’s yeast bread, others had similar effects of both breads (Figure 4). One participant obtained a reduction in overall score of almost 30 after intake of sourdough and a score increase of almost 35 after intake of baker’s yeast. Half of the participants (4 out of 8) who obtained an increase in overall score after sourdough intervention also obtained an increase after baker’s yeast. Three participants experienced no difference in diarrhoea score after neither sourdough nor baker’s yeast intervention. After the baker’s yeast intervention, 11 of 20 participants increased the diarrhoea score, 5 of 20 had a small decrease, while 4 did not have any change. One participant obtained a reduction in diarrhoea score of almost 20 after sourdough and a corresponding increase in the score of 20 after baker’s yeast (Figure 4).

The BSC scores had no significant differences, and the participants had normal stool consistency during both interventions. The BSC score was 4 (3.0–4.8) for the sourdough bread and 4 (2.0–5.0) for the baker’s yeast bread (Table 4).

### 3.4. Micronutrient Levels and Metabolic Markers

We next investigated whether the consumption of sourdough and baker’s yeast bread influenced circulating micronutrient levels and metabolic markers. The blood iron level was borderline significantly increased after the intervention with sourdough bread compared with baker’s yeast bread (*p* = 0.053) (Table 4). After consuming sourdough bread, 58% (11 out of 19) of the participants showed an increase in their iron levels with individual variation ranging from 3.93 μmol/mol to 140.81 μmol/mol. After consuming baker’s yeast bread, 7 out of 19 participants (37%) showed an increase in their iron levels, ranging from 0.51 μmol/mol to 105.54 μmol/mol. Several of the individuals had decreased iron levels after both interventions, while two individuals had increased iron levels after both interventions. There were no significant differences in selenium and zinc nor the metabolic markers (total cholesterol, triglycerides, and c-peptide) between or within any of the interventions (Table 4).

### 3.5. Targeted Gut Microbial Taxa

Overall, intervention with sourdough bread increased the abundance of the measured bacterial species (29 out of 45), while intervention with baker’s yeast decreased the abundance (28 out of 45) (Table 5). Of the 45 gut bacteria species included in the analyses, five species were significantly changed with an increase after intake of sourdough bread and a decrease after baker’s yeast bread: *Alistipes putredinis* (*p* = 0.029), *Alistipes shahii* (*p* = 0.015), *Bifidobacterium adolescentis* (*p* = 0.042), *Bifidobacterium longum* (*p* = 0.010) and *Bifidobacterium longum* sub-species (spp. Longum) (*p* = 0.021) (Table 5).

### 3.6. Correlations Between Targeted Gut Microbial Taxa, Metabolic Markers, Gut Symptoms and Micronutrient Levels

**Figure 6:**
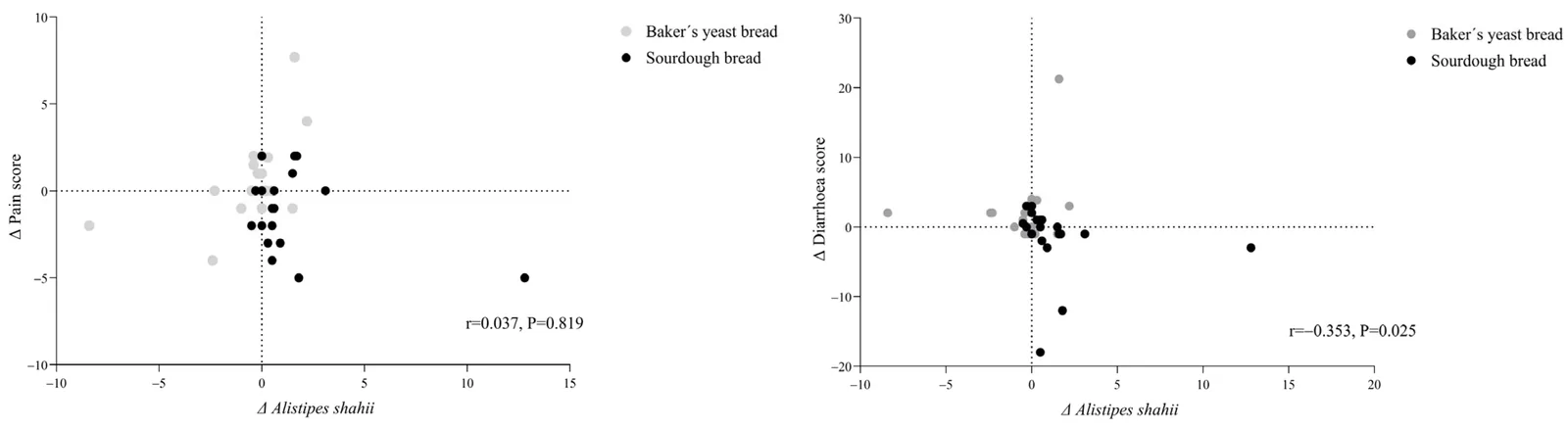
Correlation between change in pain score and diarrhoea score and change in *Alistipes shahii*. There was no significant correlation between change in pain score and change in abundance of *Alistipes shahii* (r = 0.037, *p* = 0.819), but there was a negative correlation between change in diarrhoea score and change in abundance of *Alistipes shahii* (r = −0.353, *p* = 0.025). Correlation analyses were performed with Spearman’s rho, and significance level was defined as *p* < 0.05. Figures were made with GraphPad.

**Figure 7:**
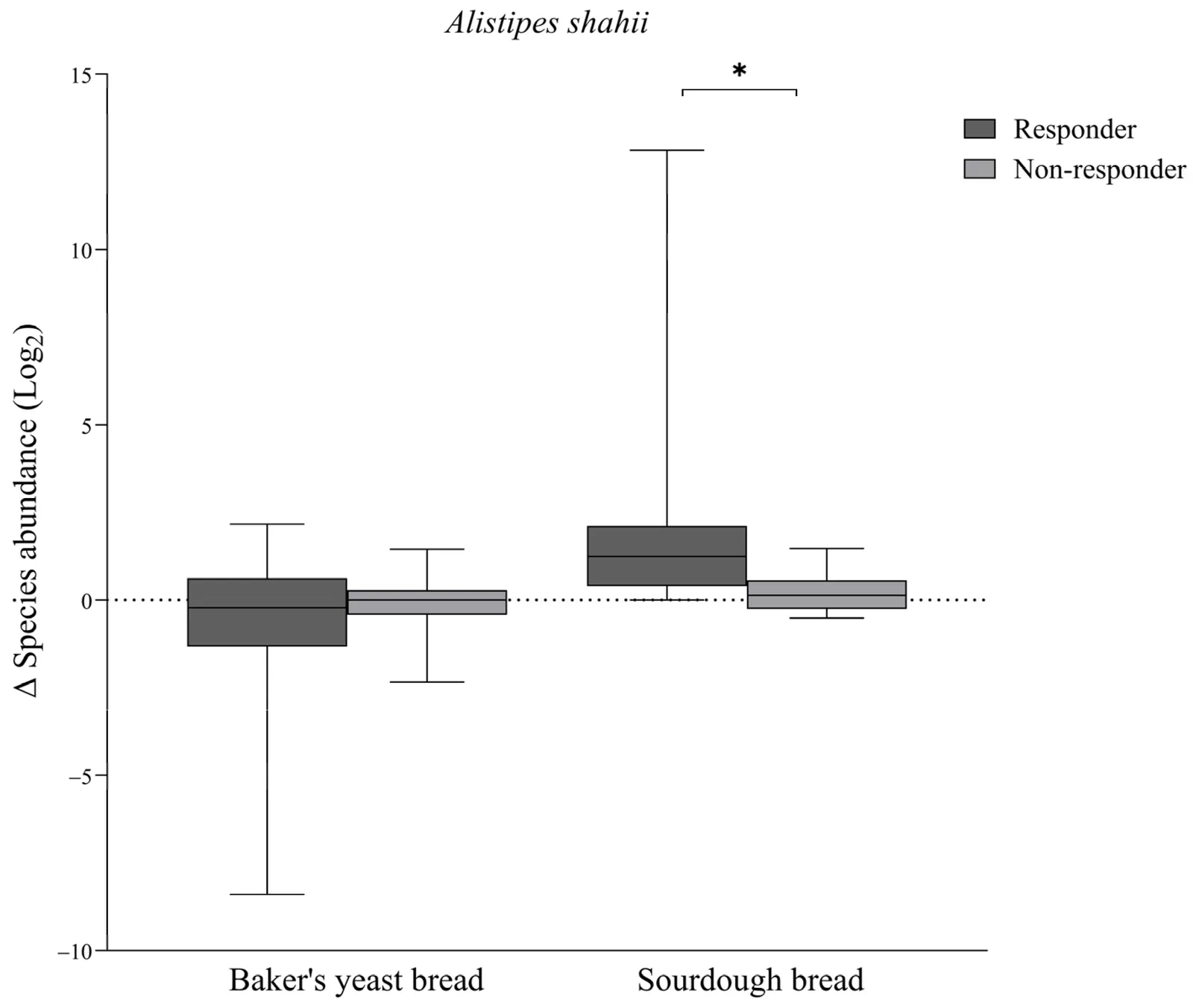
Changes in relative abundance of *Alistipes shahii* between responders and non-responders. Responders were identified by scores below median GSRS-IBS diarrhoea after the sourdough bread intervention compared to non-responders (scores above median GSRS-IBS diarrhoea). Statistical differences between responders and non-responders were analysed using the Mann–Whitney test, and significance level was defined as *p* < 0.05. * Indicates *p*-value < 0.05. The figure was made with GraphPad.

We further analysed the correlation between the significantly changed abundance of the selected gut bacteria species and changes in GSRS-IBS (Overall GSRS-IBS, pain, bloating, constipation, diarrhoea, satiety), BSC, biomarkers for metabolic regulation (triglycerides, c-peptide, total cholesterol) and micronutrient levels (iron, zinc, selenium) after intake of sourdough versus baker’s yeast bread (Figure 5). For the sourdough bread intervention, we found a significant negative correlation between *Alistipes shahii* and diarrhoea (r = −0.581, *p* = 0.007). For the baker’s yeast bread intervention, we found GSRS-IBS pain to be positively correlated with *Alistipes shahii* (r = 0.468, *p* = 0.037) and with *Alistipes putredinis*, although this correlation was not statistically significant (r = 0.438, *p* = 0.053). Lastly, *Bifidobacterium adolescentis* were negatively correlated with iron (r = −0.447, *p* = 0.055) and zinc (r = −0.445, *p* = 0.056) but did not reach statistical significance (Figure 5). When analysing the correlation between changes in pain and diarrhoea scores and change in the abundance of *Alistipes shahii*, independent of interventions, the correlation between diarrhoea and *Alistipes shahii* remained significant (r = −0.353, *p* = 0.025) but not pain score (Figure 6). The only gut symptom with a beneficial significant effect after sourdough intervention and a correlation with a bacterium (*Alistipes shahii*) was diarrhoea. Therefore, we divided the participants who achieved a reduction in diarrhoea score (below median score) (responders, n = 10) after sourdough bread with those who did not (above median score) (non-responders, n = 10) and investigated the abundance of *Alistipes shahii* between the responders and non-responders. The responders had a significantly higher abundance of *Alistipes shahii* after sourdough intervention compared with the non-responders (*p* = 0.015). There was no difference between responders and non-responders after intervention with baker’s yeast bread (Figure 7).

## 4. Discussion

In this study, both intervention breads were made of whole grain wheat and were made from identical recipes except for the leavening agent (sourdough vs. baker’s yeast) and leavening duration. Although the participants were healthy without diagnosis of gut disease and they had a high intake of fibre in their habitual diet, they obtained some non-significant beneficial effects related to gut symptoms after the intake of sourdough bread compared to baker’s yeast bread. The intake of sourdough bread reduced overall gut symptoms and diarrhoea and increased the abundance of *Bifidobacterium subspecies* (spp.) and *Alistipes* spp. There were some correlations between selected gut bacterial species and gut symptoms. When dividing the participants into responders and non-responders of sourdough bread on diarrhoea score, there was a significant increase in the abundance of *Alistipes shahii* in the responders. Circulating iron levels showed a trend towards being increased after intervention with sourdough bread compared to baker’s yeast bread. There were no significant effects of sourdough bread compared to baker’s yeast bread on circulating levels of zinc, selenium, c-peptide, triglycerides and total cholesterol.

In this study, the sourdough bread caused a reduction in the overall GSRS-IBS score and the GSRS-IBS diarrhoea sub-dimension score compared with baker’s yeast bread. Previous studies investigating the effects of sourdough bread consumption on gastrointestinal symptoms have shown that the FODMAPs content may affect gut symptoms [21,22]. For instance, Laatikainen et al. found that IBS patients experienced significantly milder flatulence, cramps and rumbling when consuming a low FODMAPs rye sourdough bread compared to rye sourdough bread with a higher content of FODMAPs [21]. These effects were found after consuming 0.45 g fructans and 0.60 g total FODMAPs per day in the low FODMAPs group compared to 1.73 g fructans and 2.21 g total FODMAPs in the high FODMAPs group.

This is not in line with the present study, where there was a higher amount of fructans in the sourdough bread (1.60 g/day) than in the baker’s yeast bread (0.80 g/day). Unfortunately, total FODMAPs were not measured in the breads of this study.

In a review by Varney et al., they compare excess fructose, GOS and fructan content in two slices of common bread from Australia, USA and Norway [33]. In the wholegrain wheat bread from Norway (80 g), the total amount of fructans was approximately 1.5 g and there was no excess fructose. In this paper, the content of fructans in the breads was lower than reported by Varney et al. (Table 3). The cut-off value for low FODMAPs in grains/cereals is <0.15 g excess fructose per serving [33]. The content of fructose was higher than that of glucose in the baker’s yeast bread (1.74g fructose/d and 0.60 g glucose/d), but it was lower in the sourdough bread (0.22 g fructose/d and 0.72 g glucose/d) (Table 3).

Fructose malabsorption is common among individuals who experience gut symptoms [34]. Poorly absorbed fructose can trigger gut symptoms and lead to osmotic diarrhoea. Reducing fructose in the diet can alleviate these symptoms [35]. Therefore, the decreased fructose intake from the sourdough bread in this study might have contributed to the reduction in overall gut symptoms and diarrhoea. This is in line with the literature showing that bread after prolonged fermentation does not have fructose in excess of glucose [11,17]. However, the breads also differed in fructans, LMWDF, sugar profile and fermentation metabolites, which may have effects on gut symptoms. Although the breads in this study may not be defined as low FODMAP, we did observe some beneficial effects of the sourdough bread on gut symptoms, which may be due to the low fructose content.

Studies have shown that sourdough bread could influence the intestinal microbiota by supporting the growth of beneficial bacteria [36,37]; however, this is yet to be consistently demonstrated [18]. In this study, when performing secondary endpoint analyses, we found that an intake of sourdough bread increased the abundance of *Alistipes putredinis* and *Alistipes shahii*, in addition to three species of the *Bifidobacterium* genus, compared with intake of baker’s yeast bread. The abundance of short-chain fatty acids (SCFAs)-producing bacteria, such as *Bifidobacterium* spp., has in several previous studies shown to be affected by changes in diet [38,39,40,41,42,43,44]. In particular, prebiotics like FOS and GOS are known to promote the growth of *Bifidobacterium* [4]. In this study, there was little difference in the content of fructans (FOS/inulin) between the study breads, and the GOS content of the breads was not measured. Whether the observed increase in *Bifidobacterium* is caused by differences in prebiotics between the breads is therefore not known.

The administration of some probiotics, particularly *Bifidobacterium* spp., is shown to successfully moderate gastrointestinal symptoms, predominantly abdominal pain, diarrhoea, and bloating [45,46,47,48]. For instance, supplementation with *Bifidobacterium longum* subsp. *longum* has previously been shown to significantly reduced diarrhoea incidents [49,50]. It was therefore interesting that *Bifidobacterium adolescentis, Bifidobacterium longum* and *Bifidobacterium longum* subsp. *longum* were increased, although there was no significant correlation between *Bifidobacterium* and gut symptoms. We did find a significant positive correlation between *Alistipes shahii* and pain score (after baker’s yeast intervention) and a significant negative correlation between *Alistipes shahii* and diarrhoea score (after sourdough intervention) (Figure 5 and Figure 6). These results are interesting considering *Alistipes shahii* is regarded as an important bacterium in the human gut and is associated with gut diseases [51]. However, these exploratory correlations require independent confirmation. It has been shown that patients with inflammatory bowel disease (IBD) have lower levels of *Alistipes shahii* in the gut [52,53]. This is partly in line with our findings, showing a correlation between higher *Alistipes shahii* levels and lower diarrhoea score but higher pain score. When dividing the group into responders and non-responders of sourdough bread on diarrhoea, we found a significant higher abundance of *Alistipes shahii* in the responders compared to the non-responders (Figure 7). Laatikainen et al. found that responders and non-responders had a significantly different abundance of specific bacteria at baseline, but there was no significant change in bacteria abundance after interventions with high or low FODMAP [54].

We observed that circulating levels of iron showed a strong trend (*p* = 0.053) towards being increased after intervention with sourdough bread compared to baker’s yeast bread. It is well documented that phytic acid in bread can inhibit iron absorption [55,56]. One study suggested that prolonged sourdough fermentation may reduce phytate levels, potentially increasing iron bioavailability [57]. This paper showed no difference in the iron content between the two breads, yet the iron levels in the circulation borderline significantly increased after consuming sourdough bread but not baker’s yeast bread. Therefore, sourdough fermentation could have contributed to the breakdown of phytic acid in the bread, enabling the body to absorb more iron, as shown by others [55,58]. However, in this study, we did not observe differences in the phytic acid concentration between the two breads. When looking at the individual level, there is no consistency in changes in iron levels after the different interventions. Rather, several individuals increase their iron level after both interventions, while others decrease their iron level after both interventions. This may indicate that there are other factors affecting iron uptake than fermentation alone.

## 5. Study Strengths and Limitations

This study has both strengths and limitations. The main strength is the robust study design: it is a randomized, controlled, double blind, cross-over study. The lack of formal sample size calculations and the small sample size may have resulted in reduced statistical power, potentially affecting the results. However, it is worth noting that cross-over designs typically require fewer participants to achieve statistical power, since each participant serves as their own control. Additionally, mean values were employed to replace missing values in the dataset, which presents another limitation. The participants in this study were included based on self-reported gut symptoms after bread consumption but were otherwise healthy. Hence, the observed effects are limited to those without known diseases and may reflect considerable individual differences. We conducted targeted gut microbiota analysis, which may have both strengths and limitations. Strengths include a high sensitivity and specificity of the given bacteria, while a limited overview of the microbiome is a limitation. Participants were randomly allocated to begin with the sourdough bread or the baker’s yeast bread, avoiding any biases of period effect during the study. Because this study was double-blind, participants and researchers were unaware of the randomisation order, reducing the risk of systematic bias and the placebo effect. Blinding is limited because this dietary intervention study included two types of bread, which differed in taste and appearance (sourdough and baker’s yeast breads). Consequently, some participants may have identified the type of bread they were consuming, causing them to be influenced by their expectations towards that specific bread. No major self-reported changes in diet, physical activity, or health status during the study period were identified, although residual confounding cannot be excluded. It is important to note that this study was not a fully controlled dietary intervention study, and we cannot discount the possibility of other dietary factors influencing the results.

## 6. Conclusions

Even though sourdough bread may cause fewer gut symptoms, this exploratory randomized, controlled, double-blind cross-over study did not demonstrate a statistically significant difference in the overall GSRS-IBS score between sourdough bread and baker’s yeast bread. A nominal difference in the diarrhoea sub-dimension and selected targeted microbial taxa was observed between the two breads, but these findings require further investigations in larger studies.

## Figures and Tables

**Table 1:** Baseline characteristics.

	Median	25–75th Percentiles
Total participants (n)	20	
Males/Females	5/15	
Age (years)	28	21–44
Height (cm)	169	160.3–180.8
Weight, (kg)	65	60.3–71.8
BMI (kg/m^2^)	22.2	20–24.7
HbA1c (mmol/mol) ^1^	32.2	30.2–37
Total cholesterol (mmol/L) ^1^	4.6	3.8–5.2
Triglycerides (mmol/L) ^1^	0.9	0.7–1.4
C-peptide (pmol/L) ^1^	298.8	229.5–336.6

HbA1c, total cholesterol, triglycerides and c-peptide are measured at e-v1 in fasting capillary blood ^1^ (n = 19). Abbreviations: BMI; Body mass index, e-v1; e-visit 1.

**Table 2:** Macronutrient intake assessed by food frequency questionnaire (FFQ) (n = 19).

	Median Gram/Day	25–75th Percentiles	Energy (%)/Day
Energy (kJ)	9227	8059–10,836	
Carbohydrate	240.5	199.4–300.5	42.4
Dietary fibre	29.5	20.2–39.5	2.1
Protein	90.8	69.5–111.0	15.6
Fat	85.9	71.1–118.6	35.0
Polyunsaturated fat	16.4	12.7–24.6	6.5
Saturated fat	32.3	24.2–42.4	12.4
Alcohol	9.0	0.6–17.2	3.1
Bread consumption	102.3	52.9–170.0	
Whole grain	53.3	27.1–76.9	

Data are given as median with 25–75th percentiles. Abbreviations: kJ; kilojoule.

**Table 3:** Nutritional composition of the study breads.

	Baker’s Yeast Bread		Sourdough Bread	
	Per 100 g	Per Day (200 g)	Per 100 g	Per Day (200 g)
Energy kJ (kcal)	924 (221)	1848 (442)	887 (212)	1774 (424)
Fat (total) (g)	1.57	3.14	1.05	2.10
Saturated fat (g) ^1^	0.41	0.82	0.25	0.50
Monosaturated fat (g) ^1^	0.35	0.70	0.23	0.46
Polyunsaturated fatty acids (g) ^1^	0.69	1.38	0.49	0.98
Protein (g)	9.50	19.00	10.10	20.20
Carbohydrates (g)	38.40	76.80	36.80	73.60
Sugars (g) ^1^	2.83	5.66	1.84	3.68
**Fructose (g) ^1^**	**0.87**	**1.74**	**0.11**	**0.22**
Glucose (g) ^1^	0.30	0.60	0.36	0.72
**Lactose (g) ^1^**	**<0.04**	**<0.08**	**<0.04**	**<0.08**
Maltose (g) ^1^	1.54	3.08	1.23	2.46
Sucrose (g) ^1^	0.12	0.24	0.14	0.28
Galactose (g) ^1^	<0.04	<0.016	<0.04	<0.08
**Fructans (Inulin/FOS) (g)**	**0.40**	**0.80**	**0.80**	**1.60**
**Sum analysed FODMAPs ^2^**	**1.31**	**2.62**	**0.95**	**1.90**
Total dietary fibre (g)	6.50	13.00	6.30	12.60
LMWDF (g) ^1^	0.90	1.80	1.30	2.60
Total amount of ISDF (g) ^1^	4.30	8.60	3.70	7.40
Total amount of SDF (g) ^1^	1.30	2.60	1.30	2.60
Salt (g)	1.03	2.06	0.98	1.96
Phytic acid (%)	<0.14	<0.28	<0.14	<0.28
Iron (mg)	1.60	3.20	1.70	3.40
Zinc (mg)	1.30	2.60	1.20	2.40
Selenium (mg)	<0.05	<0.10	<0.05	<0.10

^1^ Listed in grams out of total respective nutrient substance. ^2^ Sum FODMAPs analysed (fructose, lactose, and fructans (inulin/FOS)) (in bold). Abbreviations: FOS; fructooligosaccharides, g; grams, ISDF; insoluble dietary fibre, kcal; kilocalorie, kJ; kilojoule, LMWDF; low molar weight dietary fibre, SDF; soluble dietary fibre. Analyses conducted by Eurofins (Eurofins Food & Feed Testing Norway AS).

**Table 4:** Effect of baker’s yeast and sourdough bread on metabolic markers, satiety hormone and gut symptoms before and after intervention (n = 20).

	Baker’s Yeast Bread			Sourdough Bread			
	Pre	Post	*p*-Value ^1^	Pre	Post	*p*-Value ^1^	*p*-Value ^2^
**Gut symptoms**							
Bristol stool chart	4.00 (3.00–4.38)	4.00 (2.00–5.00)	0.563	4.00 (3.13–4.75)	4.00 (3.00–4.88)	0.835	0.777
**Metabolic markers ^3^**							
Total cholesterol (mmol/L)	4.61 (3.82–5.24)	4.50 (3.77–5.63)	0.872	4.52 (3.61–5.31)	4.59 (4.07–5.19)	0.117	0.212
Triglycerides (mmol/L)	0.94 (0.70–1.44)	1.06 (0.70–1.27)	0.904	0.82 (0.67–1.23)	0.85 (0.74–1.03)	0.904	1.00
C-peptide (pmol/L)	283.20 (252.35–336.59)	298.93 (233.88–358.14)	0.469	262.16 (210.34–319.92)	278.80 (196.26–320.04)	0.717	0.872
HbA1c (mmol/mol)	32.19 (28.16–35.49)	32.69 (31.47–35.86)	0.355	32.16 (30.43–35.41)	32.74 (28.80–35.58)	0.573	0.126
**Micronutrient levels ^3^**							
Iron (μmol/mol)	534.27 (498.48–625.60)	529.49 (491.14–581.18)	0.117	527.45 (487.88–548.32)	535.47 (492.73–592.71)	0.601	0.053
Zinc (µg/mL)	5.94 (5.21–7.56)	6.23 (4.86–6.61)	0.126	5.95 (5.49–6.84)	6.08 (5.59–7.25)	0.717	0.184
Selenium (µg/mL)	0.16 (0.13–18)	0.16 (0.14–0.17)	0.198	0.15 (0.13–0.18)	0.16 (0.12–0.18)	1.00	0.099

Data are given as median (25–75th percentiles). Pre and post indicate before and after the interventions. *p*-values < 0.05 indicate significant differences within or between groups. Differences within ^1^ and between ^2^ groups were analysed by Wilcoxon signed-rank test. ^3^ (n = 19). Abbreviations: HbA1c; haemoglobin A1c.

**Table 5:** Change in abundance of bacterial species in faeces before and after consuming sourdough bread or baker’s yeast bread (n = 20).

	Baker’s Yeast Bread				Sourdough Bread				Sourdough/Baker’s Yeast	
	Pre	Post	Post/Pre	*p*-Value ^1^	Pre	Post	Post/Pre	*p*-Value ^1^		*p*-Value ^2^
Species	Mean (SD)	Mean (SD)	Log2 ratio		Mean (SD)	Mean (SD)	Log2 ratio		Log2 ratio	
*Akkermansia muciniphila*	8.17 (5.46)	7.18 (6.18)	−0.99	ns	7.52 (5.57)	7.47 (5.66)	−0.05	ns	0.94	ns
*Alistipes putredinis*	11.08 (5.55)	10.23(6.50)	−0.85	ns	9.75 (6.34)	11.31 (5.61)	1.56	**0.017**	2.41	**0.029**
*Alistipes shahii*	9.21 (4.25)	8.73 (4.67)	−0.48	ns	8.00 (5.00)	9.28 (4.88)	1.28	0.061	1.76	**0.015**
*Anaerobutyricum halli*	4.30 (5.01)	3.02 (4.80)	−1.28	ns	2.87 (4.61)	4.19 (4.85)	1.32	ns	2.60	ns
*Anaerostipes hadrus*	9.05 (2.93)	9.29 (3.31)	0.24	ns	9.23 (3.07)	9.74 (3.05)	0.51	ns	0.27	ns
*Bacteroides caccae*	8.27 (5.07)	8.40 (5.25)	0.13	ns	6.72 (5.82)	7.93 (5.45)	1.21	ns	1.08	ns
*Bacteroides cellulosilyticus*	7.48 (4.98)	6.98 (5.65)	−0.5	ns	6.32 (5.68)	6.50 (5.70)	0.18	ns	0.68	ns
*Bacteroides dorei*	9.97 (4.53)	9.63 (5.01)	−0.34	ns	9.59 (5.02)	9.98 (4.61)	0.39	ns	0.73	ns
*Bacteroides fragilis*	5.53 (4.88)	5.90 (4.83)	0.37	ns	4.60 (5.34)	4.85 (5.14)	0.25	ns	−0.12	ns
*Bacteroides ovatus*	10.47 (3.36)	10.65 (3.60)	0.18	ns	9.68 (3.95)	10.40 (3.53)	0.72	ns	0.54	ns
*Bacteroides thetaiotaomicron*	8.32 (3.96)	8.83 (4.22)	0.51	ns	8.16 (4.66)	8.34 (4.17)	0.18	ns	−0.33	ns
*Bacteroides uniformis*	14.65 (1.88)	14.23 (3.88)	−0.42	ns	14.46 (2.49)	14.15 (3.78)	−0.31	ns	0.11	ns
*Bacteroides vulgatus*	11.65 (4.52)	11.30 (5.19)	−0.35	ns	11.28 (4.69)	11.08 (5.18)	0.20	ns	0.55	ns
*Bacteroides xylanisolvens*	5.36 (4.82)	4.89 (5.24)	−0.47	ns	4.29 (5.07)	5.20 (5.27)	0.91	ns	1.38	ns
*Barnesiella intestinihominis*	8.13 (5.22)	7.85 (5.57)	−0.28	ns	7.30 (5.65)	7.96 (5.58)	0.66	ns	0.94	ns
*Bifidobacterium adolescentis*	5.99 (5.06)	4.90 (5.28)	−1.09	ns	4.60 (5.27)	5.18 (5.28)	0.58	ns	1.67	**0.042**
*Bifidobacterium longum*	9.33 (3.05)	8.58 (3.85)	−0.75	ns	8.01 (4.42)	8.82 (3.92)	0.81	**0.033**	1.56	**0.010**
*Bifidobacterium longum subsplongum*	9.58 (3.04)	9.00 (3.75)	−0.58	ns	8.35 (4.56)	9.04 (4.42)	0.69	0.059	1.27	**0.021**
*Bifidobacterium pseudocatenulatum*	4.98 (5.01)	4.85 (5.10)	−0.13	ns	4.95 (4.63)	4.50 (5.44)	−0.45	ns	−0.32	ns
*Bilophila wadsworthia*	7.52 (3.49)	6.36 (4.39)	−1.16	ns	6.47 (4.06)	6.40 (4.36)	−0.07	ns	1.09	ns
*Citrobacter koseri*	2.67 (4.76)	3.48 (5.14)	0.81	ns	0.00 (0.00)	3.43 (5.44)	3.43	ns	2.62	ns
*Clostridium leptum*	3.69 (2.60)	3.16 (2.24)	−0.53	ns	3.03 (2.94)	3.25 (2.82)	0.22	ns	0.75	ns
*Collinsella aerofaciens*	10.49 (1.31)	9.92 (3.66)	−0.57	ns	9.46 (3.48)	9.75 (3.61)	0.29	ns	0.86	ns
*Coprococcus catus*	8.50 (3.04)	8.01 (3.64)	−0.49	ns	8.52 (3.11)	8.17 (3.79)	−0.35	ns	0.14	ns
*Coprococcus comes*	8.71 (2.42)	8.64 (2.62)	−0.07	ns	8.37 (2.51)	8.44 (2.43)	0.07	ns	0.14	ns
*Dorea formicigenerans*	7.44 (1.96)	7.21 (2.68)	−0.23	ns	7.40 (2.01)	6.52 (2.87)	−0.88	ns	−0.65	ns
*Dorea longicatena*	9.63 (2.45)	9.51 (2.70)	−0.12	ns	9.39 (2.40)	9.44 (2.62)	0.05	ns	0.17	ns
*Eubacterium eligens*	5.71 (4.98)	7.38 (4.78)	1.67	ns	5.88 (5.08)	6.37 (5.00)	0.49	ns	−1.18	ns
*Eubacterium rectale*	12.34 (2.34)	11.91 (3.59)	−0.43	ns	12.05 (2.28)	12.05(2.17)	0.00	ns	0.43	ns
*Eubacterium siraeum*	6.45 (4.06)	5.29 (5.04)	−1.16	ns	6.11 (4.41)	5.36 (4.67)	−0.75	ns	0.41	ns
*Eubacterium ventriosum*	5.43 (4.82)	5.42 (4.86)	−0.01	ns	5.39 (4.90)	4.36 (4.56)	−1.03	ns	−1.02	ns
*Faecalibacterium prausnitzii*	9.31 (4.30)	9.51 (4.49)	0.20	ns	9.09 (4.88)	9.62 (4.59)	0.53	ns	0.33	ns
*Haemophilus parainfluenzae*	4.00 (4.32)	4.22 (4.69)	0.22	ns	4.61 (5.07)	4.18 (4.60)	−0.43	ns	−0.65	ns
*Odoribacter splanchnicus*	9.42 (3.28)	9.94 (3.66)	0.52	ns	8.87 (3.89)	8.94 (3.91)	0.07	ns	−0.45	ns
*Parabacteroides distasonis*	8.68 (4.01)	8.47 (4.48)	−0.21	ns	7.73 (4.71)	7.94 (4.87)	0.21	ns	0.42	ns
*Parabacteroides merdae*	8.10 (5.05)	7.90 (5.50)	−0.20	ns	7.54 (5.33)	8.07 (5.14)	0.53	ns	0.73	ns
*Paraprevotella clara*	3.29 (5.31)	4.05 (5.38)	0.76	ns	3.15 (5.16)	4.50 (5.32)	1.35	ns	0.59	ns
*Prevotella copri*	7.70 (6.59)	7.77 (6.04)	0.07	ns	7.49 (6.29)	7.18 (5.96)	−0.31	ns	−0.38	ns
*Roseburia hominis*	9.40 (1.48)	8.83 (2.60)	−0.57	ns	7.28 (4.49)	7.13 (4.28)	−0.15	ns	0.42	ns
*Roseburia intestinalis*	6.99 (4.45)	6.45 (4.80)	−0.54	ns	7.06 (4.18)	6.51 (4.37)	−0.55	ns	−0.01	ns
*Roseburia inulinivorans*	7.62 (4.36)	8.29 (4.19)	0.67	ns	7.94 (4.16)	7.73 (4.41)	−0.21	ns	−0.88	ns
*Ruminococcus bromii*	6.03 (6.24)	6.09 (6.35)	0.06	ns	6.31 (5.84)	6.23 (6.10)	−0.08	ns	−0.14	ns
*Ruminococcus torques*	4.50 (4.80)	4.87 (4.89)	0.37	ns	4.40 (5.13)	3.79 (4.58)	−0.61	ns	−0.98	ns
*Streptococcus thermophilus*	1.90 (3.10)	1.92 (3.16)	0.02	ns	2.55 (3.70)	2.14 (3.63)	−0.41	ns	−0.43	ns
*Sutterella wadsworthensis*	5.97 (5.85)	6.55 (6.02)	0.58	ns	5.98 (5.89)	7.42 (5.70)	1.44	ns	0.86	ns

Values are presented as mean and standard deviation. Pre and post indicate before and after the interventions. Values are Log2 transformed, and differences within and between interventions are presented as Log2 ratio post/pre or Log2 ratio sourdough/baker’s yeast bread, respectively. Differences within ^1^ and between ^2^ interventions were analysed by paired samples *t*-test. *p*-values < 0.05 indicate significant differences. Significant difference within and between interventions marked as bold. Abbreviations: ns: not significant; SD: standard deviation.

## Data Availability

The datasets presented in this article are not readily available because of privacy restrictions. Requests to access the datasets should be directed to corresponding author.

## Abbreviations

BMI	Body mass index
BSC	Bristol stool chart
CRF	Case report form
DBS	Dried blood spots
ECL	Electrochemiluminescence
ELISA	Enzyme-linked immunosorbent assay
FFQ	Food frequency questionnaire
FODMAPs	Fermentable oligo-, di-, monosaccharides, and polyols
FOS	Fructooligosaccharides
g	Grams
GC-FID	Gas chromatography–flame ionisation detection
GOS	Galactooligosaccharides
GSRS-IBS	Gastrointestinal symptom rating scale for irritable bowel syndrome
IBD	Irritable bowel disease
IBS	Irritable bowel syndrome
ISDF	Insoluble dietary fibre
Kcal	Kilocalorie
kJ	Kilojoule
LC	Liquid chromatography
LC-UV	Liquid chromatography with ultraviolet detection
LLOQ	Lower limit of quantitation
LMWDF	Low molar weight dietary fibre
Log2	Logarithmically transformed with base 2
MCP-1	Monocyte chemoattractant protein-1
Nm	Nanometres
OsloMet	Oslo Metropolitan University
SCFA	Short-chain fatty acids
SDF	Soluble dietary fibre
SD	Standard deviation
Spp	Sub-species
